# A Web-Based Prediction Model for Cancer-Specific Survival of Elderly Patients With Early Hepatocellular Carcinoma: A Study Based on SEER Database

**DOI:** 10.3389/fpubh.2021.789026

**Published:** 2022-01-13

**Authors:** Taiyu He, Tianyao Chen, Xiaozhu Liu, Biqiong Zhang, Song Yue, Junyi Cao, Gaoli Zhang

**Affiliations:** ^1^Key Laboratory of Molecular Biology for Infectious Diseases, Ministry of Education, Chongqing Medical University, Chongqing, China; ^2^Institute for Viral Hepatitis, Chongqing Medical University, Chongqing, China; ^3^Department of Infectious Diseases, The Second Affiliated Hospital of Chongqing Medical University, Chongqing, China; ^4^College of Medical Informatics, Chongqing Medical University, Chongqing, China; ^5^Department of Cardiology, The Second Affiliated Hospital of Chongqing Medical University, Chongqing, China; ^6^Department of Gynecology and Obstetrics, The Second Affiliated Hospital of Chongqing Medical University, Chongqing, China; ^7^Department of Record Room, Zigong First People's Hospital, Zigong, China

**Keywords:** nomogram, elderly patients, early HCC, cancer-specific survival, SEER database, online application

## Abstract

**Background:** Primary liver cancer is a common malignant tumor primarily represented by hepatocellular carcinoma (HCC). The number of elderly patients with early HCC is increasing, and older age is related to a worse prognosis. However, an accurate predictive model for the prognosis of these patients is still lacking.

**Methods:** Data of eligible elderly patients with early HCC in Surveillance, Epidemiology, and End Results database from 2010 to 2016 were downloaded. Patients from 2010 to 2015 were randomly assigned to the training cohort (*n* = 1093) and validation cohort (*n* = 461). Patients' data in 2016 (*n* = 431) was used for external validation. Independent prognostic factors were obtained using univariate and multivariate analyses. Based on these factors, a cancer-specific survival (CSS) nomogram was constructed. The predictive performance and clinical practicability of our nomogram were validated. According to the risk scores of our nomogram, patients were divided into low-, intermediate-, and high-risk groups. A survival analysis was performed using Kaplan–Meier curves and log-rank tests.

**Results:** Age, race, T stage, histological grade, surgery, radiotherapy, and chemotherapy were independent predictors for CSS and thus were included in our nomogram. In the training cohort and validation cohort, the concordance indices (C-indices) of our nomogram were 0.739 (95% CI: 0.714–0.764) and 0.756 (95% CI: 0.719–0.793), respectively. The 1-, 3-, and 5-year areas under receiver operating characteristic curves (AUCs) showed similar results. Calibration curves revealed high consistency between observations and predictions. In external validation cohort, C-index (0.802, 95%CI: 0.778–0.826) and calibration curves also revealed high consistency between observations and predictions. Compared with the TNM stage, nomogram-related decision curve analysis (DCA) curves indicated better clinical practicability. Kaplan–Meier curves revealed that CSS significantly differed among the three different risk groups. In addition, an online prediction tool for CSS was developed.

**Conclusions:** A web-based prediction model for CSS of elderly patients with early HCC was constructed and validated, and it may be helpful for the prognostic evaluation, therapeutic strategy selection, and follow-up management of these patients.

## Introduction

As the sixth most common cancer in the world, primary liver cancer is the third leading cause of cancer-related death (1). Hepatocellular carcinoma (HCC) accounts for 75% of all liver cancer cases (2). The incidence of liver cancer in elderly people is increasing (3), possibly because of an increase in life expectancy and the use of antiviral treatments (4, 5). Meanwhile, a larger proportion of patients with HCC are diagnosed at an early stage (6), possibly owing to the progress in diagnostic technology and implementation of routine screening for high-risk patients. Compared with young HCC patients, the prognosis of elderly HCC patients is worse (7–9). Consequently, accurate prognostic predictions for elderly patients with early HCC are required and may help clinicians make better decisions. Although several liver cancer staging models exist, such as the American Joint Committee on Cancer (AJCC) TNM staging system and Barcelona Clinic Liver Cancer (BCLC) staging system, they are often applied to the prognostic evaluation of all HCC stages. An accurate predictive model for the survival of elderly patients with early HCC is still lacking.

The Surveillance, Epidemiology, and End Results (SEER) database is an authoritative cancer database in the United States (10). Covering about approximately 35% of the U.S. population, it contains data that are related to cancer prognosis, such as age (11), race (12), marital status (13), histological grade (14), tumor size (15), surgery (16), chemotherapy (17), etc. A nomogram is a reliable predictive model that can accurately calculate and predict individual survival by integrating cancer-related prognostic factors (18, 19). Based on the SEER database, we tried to construct and validate a web-based prediction model for the survival of elderly patients with early HCC, which might be helpful for the prognostic prediction, treatment strategy selection and follow-up management of these patients.

## Patients and Methods

### Patients and Variables Inclusion

We used SEER^*^Stat software (version 8.3.8) to download data of HCC patients between 2010 and 2016 from the SEER database. These data contained baseline demographics, tumor features, therapeutic modalities, and survival time. The inclusion criteria were as follows: (1) age ≥65 years; (2) International Classification of Diseases for Oncology, 3rd Edition [ICD-O-3] code 8170 to 8175; (3) T1/T2, N0, and M0; and (4) follow up for 5 years or until death. The exclusion criteria were as follows: (1) unknown race; (2) unknown marital status; (3) unknown histological grade; (4) survival time <1 month; and (5) unknown whether surgery/radiotherapy was performed. Eventually, 1985 eligible elderly patients with early HCC were included in this study. The following variables were analyzed: age, race, sex, TNM stage, histological grade, tumor size, surgery, radiotherapy, chemotherapy and marital status. In addition, we used the seventh edition of the AJCC TNM staging system, which was available between 2010 and 2015.

### Statistical Analysis

For nomogram construction and validation, we randomly assigned 70% (*n* = 1093) of patients from 2010 to 2015 and 30% (*n* = 461) of patients from 2010 to 2015 to the training and validation cohorts, respectively. 431 patients from SEER database in 2016 were included in external validation cohort. Through univariate and multivariate Cox proportional hazards regression analyses, we obtained factors that significantly affected cancer-specific survival (CSS), and the hazard ratio (HR) and their 95% confidence interval (95% CI) were recorded. To construct nomogram, we assigned score to each obtained factor according to the factor's influence degree on CSS (the value of regression coefficient in the Cox proportional hazards regression model) by using “nomogramFormula” package, and then add each score to obtain the nomogram's total score. We applied the concordance index (C-index) and receiver operating characteristic (ROC) curve to reflect the discrimination and predictive accuracy of our nomogram, and their values ranged between 0 and 1.0. 0.5 represents random possibility, and 1.0 represents perfect accuracy in predicting outcomes. We constructed calibration curves using a bootstrap approach with 1,000 resamples to compare the observed survival with the predicted survival in this study. We used decision curve analysis (DCA) to assess the clinical practicability of our nomogram. Based on the cutoff value calculated from our nomogram's total score, we divided patients into a low-risk group, intermediate-risk group, and high-risk group. We applied Kaplan–Meier curves and log-rank tests to compare patient survival between different groups. A web application for survival prediction was developed based on our nomogram.

We used SPSS software (version 24.0) to perform univariate and multivariate Cox proportional hazards regression analyses. By utilizing R software (version 4.0.2) and relevant packages (“rms,” “DynNom,” “nomogramFormula,” “survival,” “foreign,” “survivalROC,” “ggDCA,” “survminer,” “shiny”), we created the nomogram, C-indices, ROCs, calibration curves, DCA curves, Kaplan–Meier curves, and a web application. We obtained the cutoff value by X-Tile software (version 3.6.1). A two-sided *P* < 0.05 was considered statistically significant.

## Results

### Characteristics of Patients

**Figure 1:**
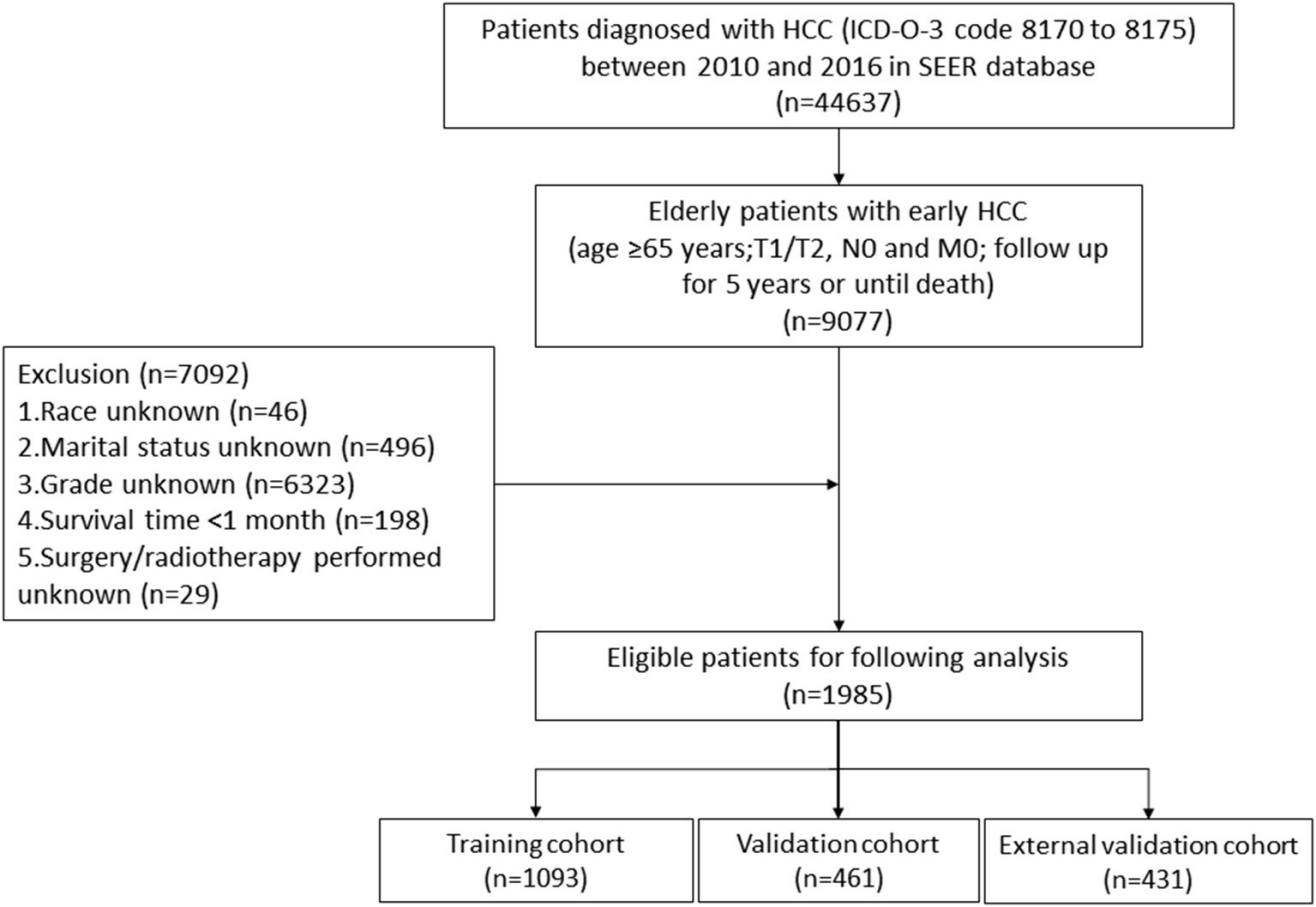
The flowchart of including and dividing patients.

Our study flowchart is shown in Figure 1. A total of 1554 eligible elderly patients with early HCC were included in our study and randomly assigned to the training cohort (*n* = 1093) and validation cohort (*n* = 461). Of these patients, 1038 (66.8%) were under 75 years old, 1050 (67.6%) were white, 1066 (68.6%) were male, 974 (62.7%) were married, 1028 (66.2%) were T1 stage, and 1332 (85.7%) were histological grade I/II. The tumors of 735 (47.3%) patients were no larger than 3 cm. A total of 1010 (65.0%) patients underwent surgery (local destruction (*n* = 342, 22.0%) and partial resection (*n* = 528, 34.0%, including wedge resection, segmental resection, lobectomy and extended lobectomy) and liver transplantation (*n* = 140, 9.0%)), while 1461 (94.0%) received radiotherapy and 488 (31.4%) received chemotherapy. Table 1 reveals that no significant difference was found in the characteristics of patients between the training cohort and validation cohort. Characteristics of patients in external validation cohort are shown in Supplementary Table S1.

**Table 1:** Patients' Characteristics.

**Variable**	**All cohort (***n*** = 1554)**	**Training cohort (***n*** = 1093)**	**Validation cohort (***n*** = 461)**	***P*** **value^d^**
	***N*** **(%)**	***N*** **(%)**	***N*** **(%)**	
Age (years)				0.511
65–74	1038 (66.8%)	724 (66.2%)	314 (68.1%)	
>74	516 (33.2%)	369 (33.8%)	147 (31.9%)	
Race				0.666
White	1050 (67.6%)	745 (68.2%)	305 (66.2%)	
Black	148 (9.5%)	100 (9.1%)	48 (10.4%)	
Other^a^	356 (22.9%)	248 (22.7%)	108 (23.4%)	
Sex				0.879
Male	1066 (68.6%)	748 (68.4%)	318 (69.0%)	
Female	488 (31.4%)	345 (31.6%)	143 (31.0%)	
Marital status				1.000
Other^b^	580 (37.3%)	408 (37.3%)	172 (37.3%)	
Married	974 (62.7%)	685 (62.7%)	289 (62.7%)	
Grade				0.675
I/II	1332 (85.7%)	940 (86.0%)	392 (85.0%)	
III/IV	222 (14.3%)	153 (14.0%)	69 (15.0%)	
T stage				0.773
T1	1028 (66.2%)	726 (66.4%)	302 (65.5%)	
T2	526 (33.8%)	367 (33.6%)	159 (34.5%)	
Tumor size (cm)				0.067
≤ 3	735 (47.3%)	500 (45.7%)	235 (51.0%)	
3–5	819 (52.7%)	593 (54.3%)	226 (49.0%)	
Surgery				0.950
No	544 (35.0%)	383 (35.0%)	161 (34.9%)	
Local destruction	342 (22.0%)	244 (22.3%)	98 (21.3%)	
Partial resection^c^	528 (34.0%)	367 (33.6%)	161 (34.9%)	
Liver transplantation	140 (9.0%)	99 (9.1%)	41 (8.9%)	
Radiotherapy				0.496
No	1461 (94.0%)	1031 (94.3%)	430 (93.3%)	
Yes	93 (6.0%)	62 (5.7%)	31 (6.7%)	
Chemotherapy				0.385
No/Unknown	1066 (68.6%)	742 (67.9%)	324 (70.3%)	
Yes	488 (31.4%)	351 (32.1%)	137 (29.7%)	

a *Other includes Asian/Pacific Islander, American Indian/Alaskan Native*.

b *Other includes single, unmarried, separated, divorced, widowed and domestic partner*.

c *Partial resection includes wedge resection, segmental resection, lobectomy and extended lobectomy*.

d *Chi-square test between training cohort and validation cohort*.

### Independent Prognostic Factors

The univariate analysis indicated that age, race, marital status, tumor size, T stage, histological grade, surgery, and chemotherapy significantly affected CSS in the training cohort (Table 2). Age, race, T stage, grade, surgery, radiotherapy, and chemotherapy were shown to be independent predictors for CSS (Table 2), and they were used to construct our nomogram.

**Table 2:** Univariate and multivariate analyses for CSS.

**Variables**	**Univariate**		**Multivariate**	
	**HR (95% CI)**	***P*** **value**	**HR (95% CI)**	***P*** **value**
Age (years)				
65–74	1 (Reference)		1 (Reference)	
>74	1.744 (1.427, 2.131)	<0.001	1.424 (1.159, 1.748)	0.001
Race				
White	1 (Reference)		1 (Reference)	
Black	1.085 (0.779, 1.511)	0.63	0.993 (0.708, 1.393)	0.967
Other^a^	0.652 (0.498, 0.852)	0.002	0.715 (0.543, 0.941)	0.017
Sex				
Male	1 (Reference)		/	
Female	1.143 (0.926, 1.412)	0.213	/	/
Marital status				
Other^b^	1 (Reference)		1 (Reference)	
Yes	0.672 (0.550, 0.821)	<0.001	0.863 (0.701, 1.061)	0.161
Grade				
I/II	1 (Reference)		1 (Reference)	
III/IV	1.32 (1.013, 1.721)	0.04	1.512 (1.156, 1.978)	0.003
T stage				
T1	1 (Reference)		1 (Reference)	
T2	1.635 (1.338, 1.997)	<0.001	1.639 (1.335, 2.013)	<0.001
Tumor size (cm)				
≤ 3	1 (Reference)		1 (Reference)	
3–5	1.297 (1.060, 1.588)	0.012	1.128 (0.913, 1.393)	0.264
Surgery				
No Surgery	1 (Reference)		1 (Reference)	
Local destruction	0.392 (0.303, 0.506)	<0.001	0.357 (0.269, 0.473)	<0.001
Partial resection^c^	0.223 (0.171, 0.290)	<0.001	0.192 (0.141, 0.260)	<0.001
Liver transplantation	0.119 (0.066, 0.213)	<0.001	0.114 (0.063, 0.208)	<0.001
Radiotherapy				
No	1 (Reference)		1 (Reference)	
Yes	0.997 (0.636, 1.564)	0.99	0.444 (0.279, 0.707)	0.001
Chemotherapy				
No/Unknown	1 (Reference)		1 (Reference)	
Yes	1.595 (1.302, 1.954)	<0.001	0.709 (0.557, 0.902)	0.005

a *Other includes Asian/Pacific Islander, American Indian/Alaskan Native*.

b *Other includes single, unmarried, separated, divorced, widowed and domestic partner*.

c *Partial resection includes wedge resection, segmental resection, lobectomy and extended lobectomy*.

*CSS, cancer-specific survival; HR, hazard ratio; CI, confidential interval*.

### Predictive Ability and Clinical Practicability of the Nomogram

**Figure 2:**
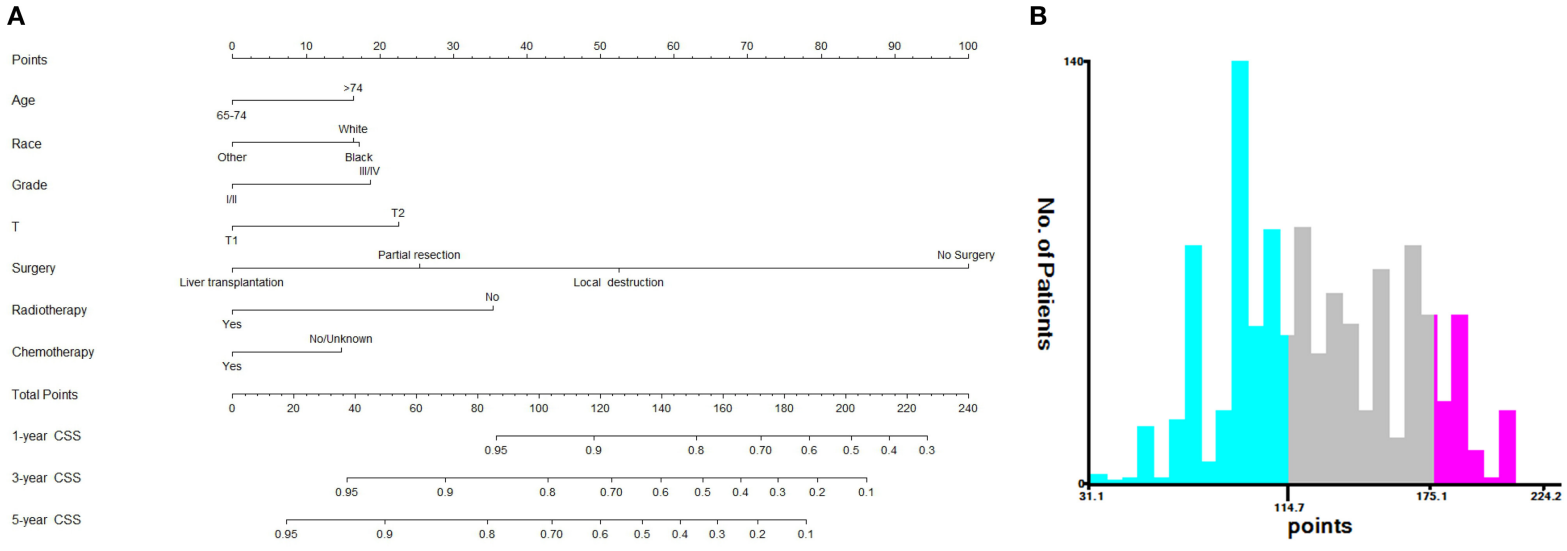
Nomogram and risk stratification model. **(A)** Nomogram for 1-, 3-, and 5-year CSS; **(B)** Risk stratification model based the nomogram.

**Figure 3:**
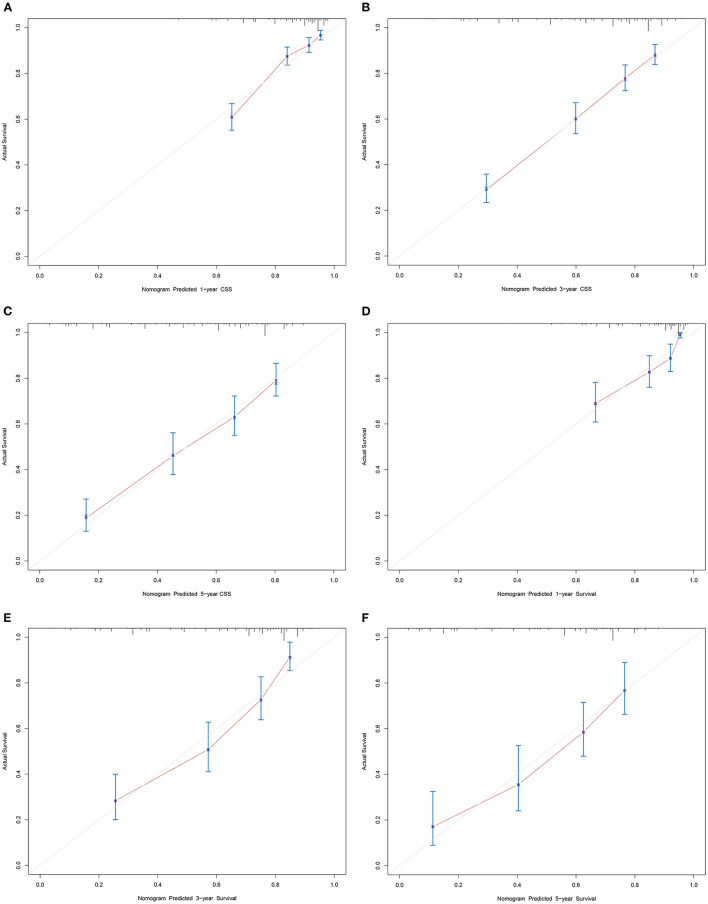
Calibration curves of nomogram. **(A–C)** For 1-, 3-, and 5-year CSS in training cohort; **(D–F)** For 1-, 3-, and 5-year CSS in validation cohort.

**Figure 4:**
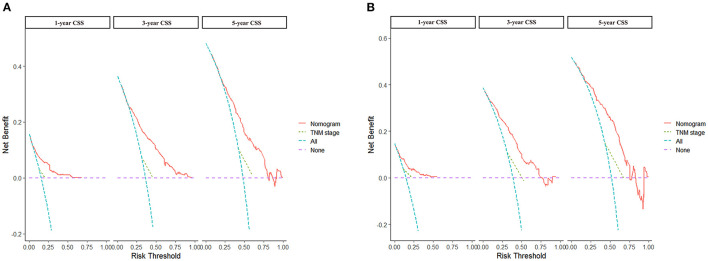
DCA curves of nomogram and AJCC staging system. **(A)** For 1-, 3-, and 5-year CSS in training cohort; **(B)** For 1-, 3-, and 5-year CSS in validation cohort.

Based on the independent prognostic factors, we constructed and validated a nomogram for predicting the 1-, 3-, and 5-year CSS of the patients (Figure 2A). In the training and validation cohorts, the C-indices of our nomogram were 0.739 (95% CI: 0.714–0.764) and 0.756 (95% CI: 0.719–0.793), respectively, while the C-indices of the AJCC TNM staging system were 0.557 (95% CI: 0.532–0.582) and 0.588 (95% CI: 0.549–0.627), respectively. Similarly, in the training cohort, the 1-, 3-, and 5-year areas under the ROC curves (AUCs) of our nomogram were 0.773, 0.779, and 0.761, respectively (Supplementary Figure S1A); and in the validation cohort, the 1-, 3-, and 5-year AUCs were 0.770, 0.790, and 0.780, respectively (Supplementary Figure S1B). For the 1-, 3-, and 5-year CSS probability, the calibration curves revealed high consistency between actual observations and nomogram predictions in both the training (Figures 3A–C) and validation (Figures 3D–F) cohorts. Moreover, compared with the TNM stage, the nomogram-related DCA curves showed better positive net benefits in both the training (Figure 4A) and validation (Figure 4B) cohorts, indicating good clinical practicability. Besides, in external validation cohort, the results of C-index (0.802, 95% CI: 0.778–0.826) and calibration curves (Supplementary Figure S2) revealed high consistency between actual observations and nomogram predictions.

### Risk Stratification System

**Figure 5:**
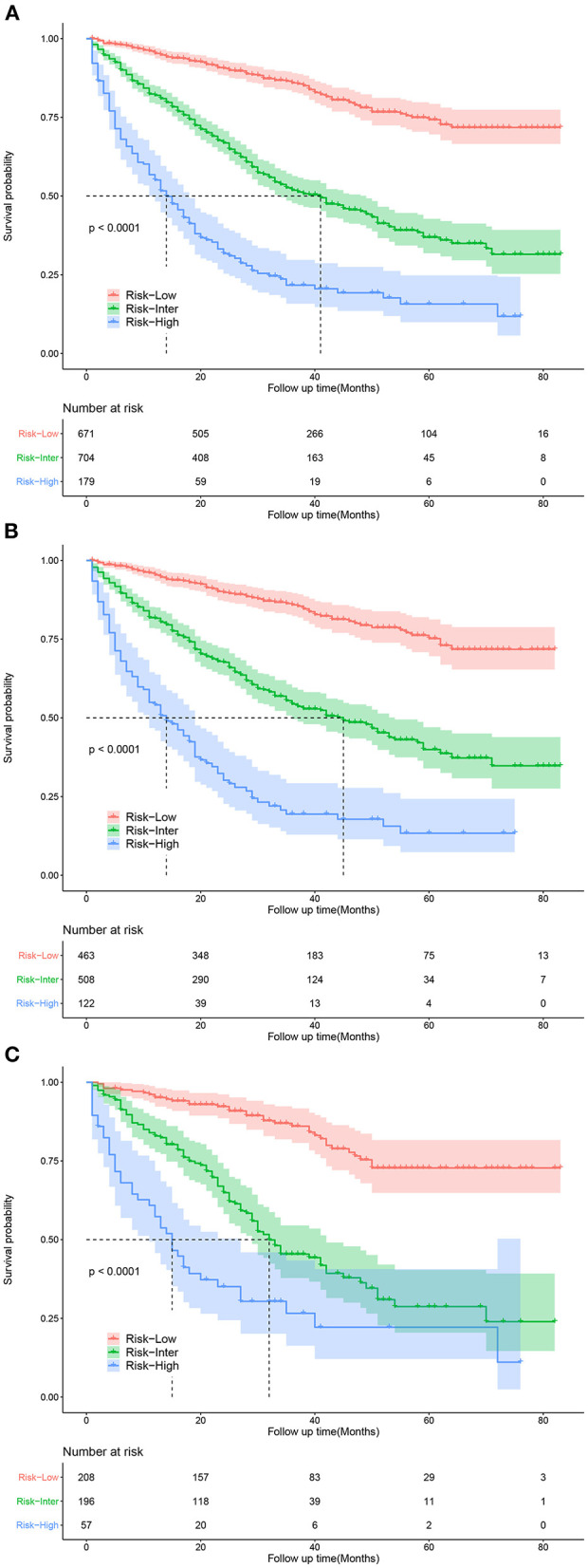
Kaplan-Meier curves for predicting CSS of patients in low-, intermediate-, and high-risk groups. **(A)** For all cohort; **(B)** For training cohort; **(C)** For validation cohort.

**Figure 6:**
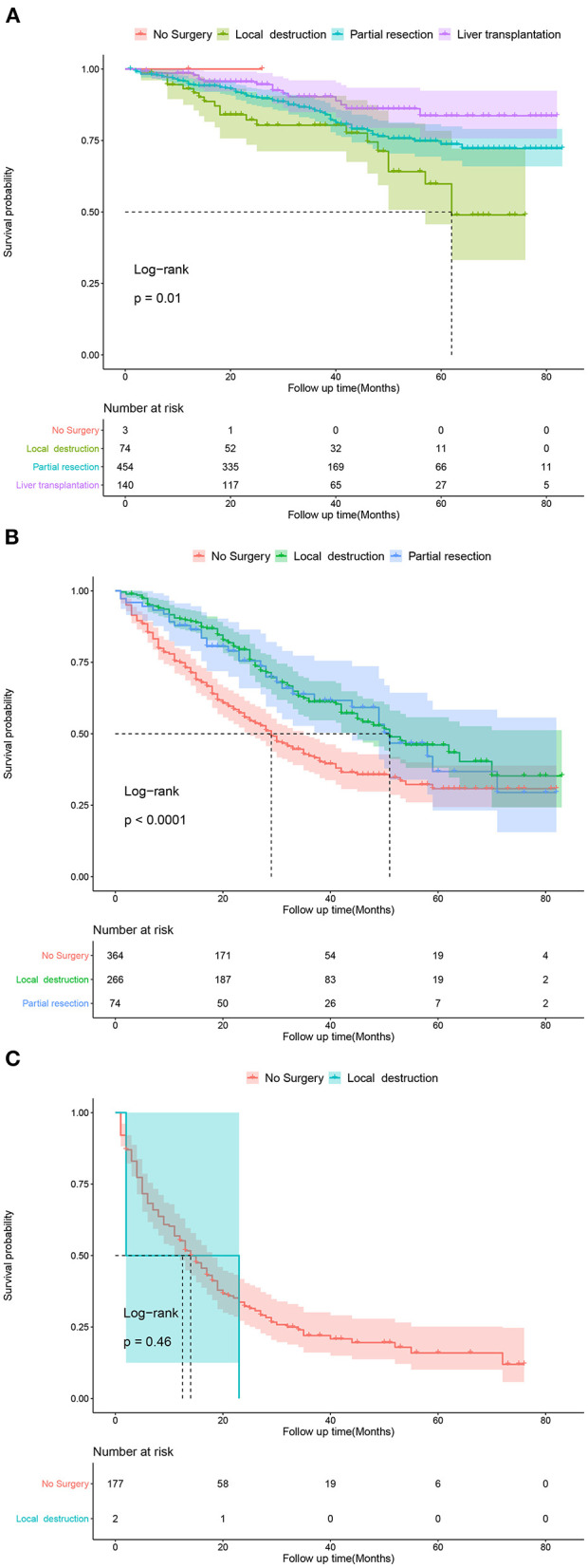
Kaplan-Meier curves predicting CSS of patients receiving different surgical methods. **(A)** In low-risk group; **(B)** In intermediate-risk group; **(C)** In high-risk group.

Based on the patients' total scores from our nomogram, we developed a risk stratification system (Figure 2B). According to the system, patients in our study were stratified into three groups: low-risk group (total score ≤ 114.7), intermediate-risk group (total score 114.7–175.1), and high-risk group (total score ≥175.1). There was a significant difference in survival probability among the three different risk groups in the all cohort, training cohort, and validation cohort (Figures 5A–C). Kaplan–Meier curves indicated that in the low-, intermediate-, and high-risk groups in all cohorts, the 1-year CSS rates were 95.3, 82.0, and 55.1%, respectively; the 3-year CSS rates were 85.9, 51.9, and 21.7%, respectively; and the 5-year CSS rates were 74.3, 36.9, and 15.7%, respectively. The 1-, 3-, and 5-year CSS rates in the training and validation cohorts were very similar to those in all cohorts. Furthermore, we compared the influences of different surgical methods on the survival probability of patients in the low-, intermediate-, and high-risk groups. In the low-risk group, patients receiving liver transplantation had the best survival probability, followed by patients receiving partial resection and then patients receiving local destruction (Figure 6A). In the intermediate-risk group, patients receiving partial resection had a similar survival probability as patients receiving local destruction, and both were higher than patients not receiving surgery (Figure 6B). In the high-risk group, no significant difference was found in survival probability between patients receiving surgery and patients not receiving surgery (Figure 6C), which was possibly due to the small sample size of patients receiving surgery.

### Online Application for CSS Prediction

Based on the nomogram, we developed an easy-to-use online application for CSS prediction, which is accessible at https://prediction-app.shinyapps.io/xiaozhuliu/. By inputting patient characteristics, the estimated survival probability can be obtained immediately, indicating that the online prediction tool is convenient for clinical use.

## Discussion

In this study, we analyzed patients' baseline demographic and clinical characteristics, and then constructed and validated a prognostic nomogram for the 1-, 3-, and 5-year CSS of elderly patients with early HCC, which may be helpful for the prognostic evaluation, treatment strategy selection, and follow-up management of these patients.

By univariate and multivariate analyses, several factors were found to significantly affect CSS, including age, race, T stage, histological grade, surgery, radiotherapy and chemotherapy. However, sex, marital status and tumor size were not independent prognostic factors, which was different from the results of Yan et al.'s study for all age groups of patients with early HCC (20), indicating that predictors for CSS of elderly patients with early HCC were different from those of other-age groups.

Among the baseline demographic variables (age, race, sex and marital status), age and race were identified as independent predictors for CSS. Previous studies revealed that younger patients had longer CSS (20, 21), which is similar to our study. Our study found that whites had longer CSS than blacks while other races (Asian/Pacific Islander, American Indian/Alaskan Native) had longer CSS than blacks and whites, which was consistent with two previous studies (21, 22), but different from others (23, 24). Although married patients with HCC were reported to have better survival (13, 20, 25), our study found that no significant difference existed in CSS between patients with different marital statuses. Consistent with previous studies, sex was not identified as a prognostic factor (20–24, 26).

For tumor feature variables (histological grade, T stage and tumor size), histological grade and T stage were identified as independent predictors for CSS in elderly patients with early HCC. We found that high T stage and poor differentiation of HCC were related to shorter CSS, which was similar to the findings of other studies (14, 20–22, 24, 25, 27). Although tumor size was recognized as an independent prognostic factor for survival in some previous studies (20, 24–26), it was not identified to significantly affect survival in our study.

Several studies have been performed on the influence of different therapeutic modalities on the prognosis of patients with early HCC. Some studies have shown that surgical resection is related to better survival (28, 29) and a lower rate of recurrence (30) compared with local destruction. Other studies revealed that surgical resection was not significantly different from other treatments in their influence on the overall survival rates (30, 31) and disease-free survival rates (31) of patients with early HCC. Older patient age was associated with less receipt of surgical therapy (16), which may be because of elderly patients' poor physical condition and relatively short remaining lifespan. In our study, liver transplantation was the most remarkable prognostic factor for CSS in all surgical methods, followed by partial resection and then local destruction. The results indicated that if conditions permitted, even traumatic surgery could be chosen as an effective therapeutic modality for elderly patients with early HCC. Although not recommended in early HCC, chemotherapy and radiotherapy were identified as beneficial to CSS in our study, indicating that elderly patients with early HCC could choose the two therapeutic modalities as effective options. Furthermore, CSS-related Kaplan–Meier curves of different surgical methods in the three risk groups were drawn. In the low-risk group, patients receiving liver transplantation had the best survival probability, followed by patients receiving partial resection and then patients receiving local destruction. In the intermediate-risk group, patients receiving partial resection had a similar survival probability as patients receiving local destruction, with both showing higher values than patients not receiving surgery. In the high-risk group, no significant difference was found in survival probability between patients receiving surgery and patients not receiving surgery, which was possibly due to the small sample size of patients receiving surgery. The above results of Kaplan–Meier curves were similar to the results of our CSS-related nomogram, which indicated that patients in the low- and intermediate-risk groups could choose surgery as an effective therapeutic modality, and if conditions permit, eligible patients in the low-risk group could choose liver transplantation as the most effective surgery. These results may be because the side effects of the treatments on elderly patients were greater considering their poor physical condition, which shortened the overall survival. These findings may help clinicians make better therapeutic decisions.

Currently, there are several staging systems for HCC, including the BCLC staging system, AJCC TNM staging system, Japan Integrated Staging (JIS) Score, Chinese University Prognostic Index (CUPI), and Cancer of the Liver Italian Program (CLIP) Score, which are usually used for prognostic evaluation of all HCC stages. In a study of 379 patients with early HCC, Nathan et al. revealed that the C-indices of the JIS, CLIP, AJCC, and BCLC staging systems were 0.52, 0.51, 0.59, and 0.51, respectively (32). In a study on 232 patients with early HCC, Santambrogio et al. revealed that for the BCLC staging system, the AUCs and C-indices were 0.5949 and 0.6479 in the training cohort and 0.5873 and 0.6323 in the validation cohort, respectively (33). Based on the SEER database, Yan et al. showed that the C-indices of the AJCC staging system for early HCC were 0.552 and 0.567 in the training and validation cohorts, respectively, which were lower than the C-indices of their nomogram (0.755 and 0.737, respectively) (20). The above studies showed that the existing staging systems did not perform very well in predicting the prognosis for some groups of HCC patients. In our study, the C-indices of the nomogram were 0.739 and 0.756 in the training and validation cohorts, respectively, which were higher than the C-indices of the AJCC staging system (0.557 and 0.588, respectively), thus indicating good discrimination and predictive accuracy. Additionally, the calibration curves of our nomogram revealed good predictive performance. The DCA curves of our nomogram had a better net benefit than those of the AJCC staging system, which implied good clinical application potential. Furthermore, Kaplan–Meier curves showed that the CSS of patients in the low-, intermediate-, and high-risk groups was significantly different from each other, indicating that our model had certain practicability. In addition, an online application for CSS prediction was constructed, which is convenient for clinical use. To the best of our knowledge, this is the first prediction model for CSS in elderly patients with early HCC based on the SEER database.

Nevertheless, this study has several deficiencies. First, this is a retrospective analysis. Second, other potential HCC-related prognostic factors, such as the etiology, HBsAg, AST and vascular invasion, were not available in the SEER database. Third, because the SEER database is mainly based on the American population, data extracted from it may not represent populations from other parts of the world.

## Conclusion

In summary, through an analysis of baseline demographic and clinical characteristics, we constructed and validated a web-based prediction model for CSS of elderly patients with early HCC, and the predictive accuracy and clinical practicability were better than those of the TNM staging system. This model is helpful for the prognostic evaluation, treatment strategy selection, and follow-up management of elderly patients with early HCC.

## Data Availability Statement

The datasets presented in this study can be found in online repositories. The names of the repository/repositories and accession number(s) can be found below: Data analyzed in this study are accessible at: https://seer.Cancer.gov/.

## Ethics Statement

Ethical review and approval was not required for the study on human participants in accordance with the local legislation and institutional requirements. Written informed consent for participation was not required for this study in accordance with the national legislation and the institutional requirements.

## Consent for Publication

All authors have reviewed the final version of the manuscript and approved its submission.

## Author Contributions

TH, TC, XL, GZ, and BZ contributed to the idea and design. XL collected and analyzed the data. XL, TH, and TC drew the figures and tables. TH and BZ wrote the draft. TH, TC, XL, BZ, SY, JC, and GZ contributed to manuscript writing and revision. All authors approved the final manuscript.

## Funding

This study was sponsored by National Science and Technology Major Project of China (2017ZX10202203 and 2018ZX10302206), the National Natural Science Foundation of China (81772198 and 81801812), and Natural Science Foundation of Chongqing, China (cstc2020jcyj-msxmX0389).

## Conflict of Interest

The authors declare that the research was conducted in the absence of any commercial or financial relationships that could be construed as a potential conflict of interest.

## Publisher's Note

All claims expressed in this article are solely those of the authors and do not necessarily represent those of their affiliated organizations, or those of the publisher, the editors and the reviewers. Any product that may be evaluated in this article, or claim that may be made by its manufacturer, is not guaranteed or endorsed by the publisher.
