# Supplementary figures and images for: A Web-Based Prediction Model for Cancer-Specific Survival of Elderly Patients With Early Hepatocellular Carcinoma: A Study Based on SEER Database
Source: Front Public Health. 2022 Jan 13;9:789026. doi: 10.3389/fpubh.2021.789026 (PMC8792840; doi:10.3389/fpubh.2021.789026)

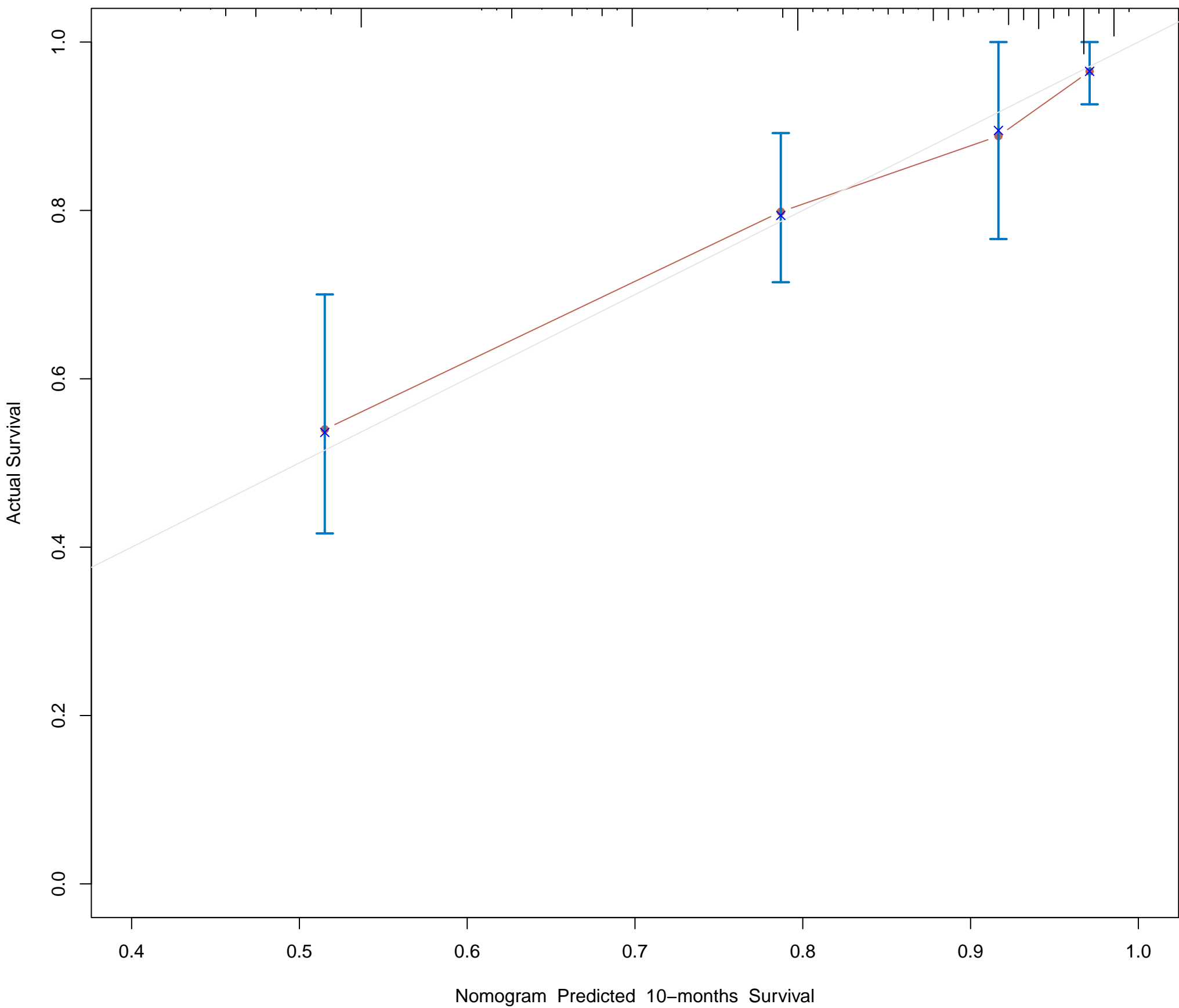

Supplement: Supplementary file 1 [file Data_Sheet_1.PDF]

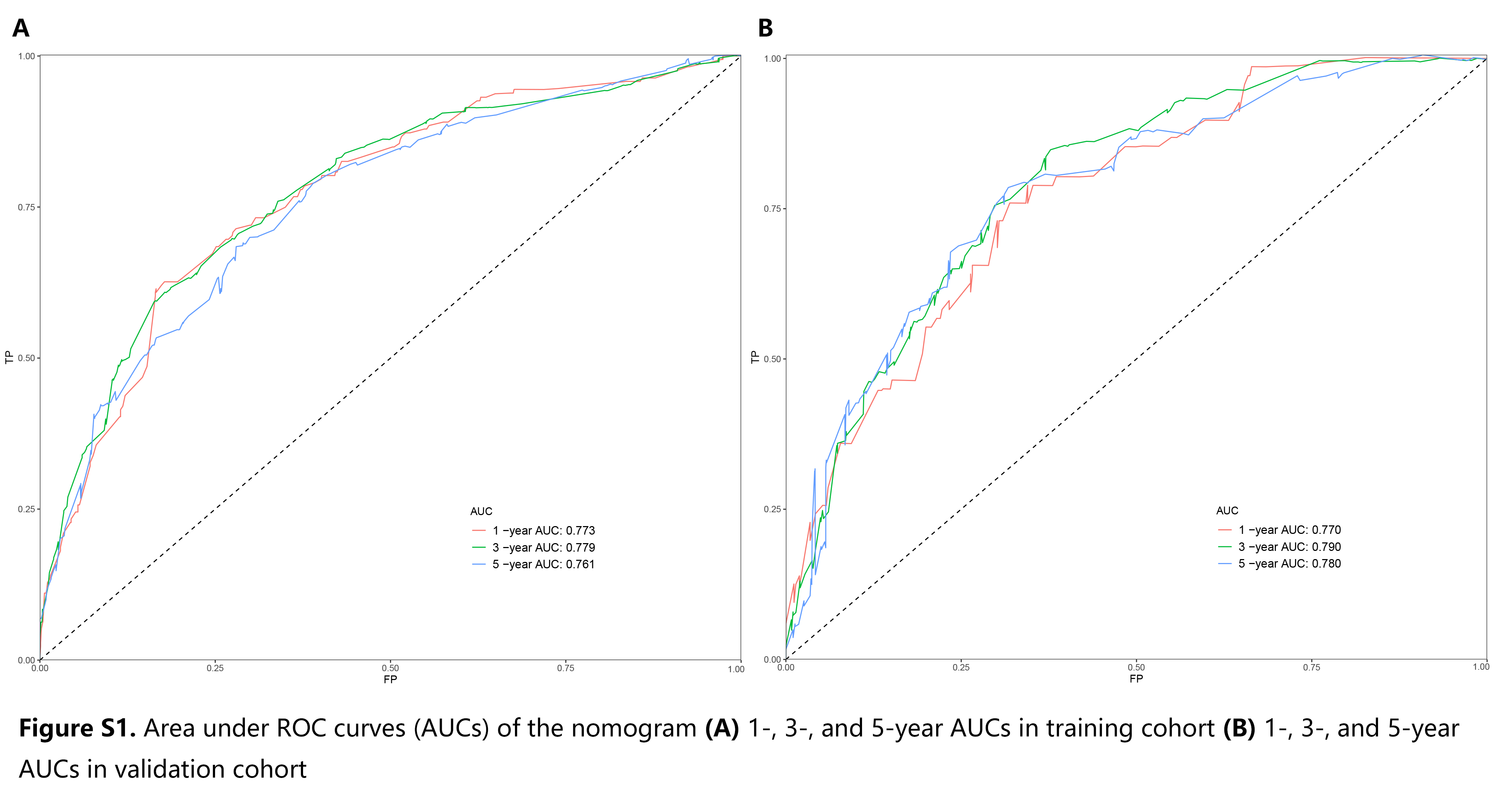

Supplement: Supplementary file 4 [file Image_1.TIF]
