# Supplementary material for: A Web-Based Prediction Model for Cancer-Specific Survival of Elderly Patients With Early Hepatocellular Carcinoma: A Study Based on SEER Database
Source: Front Public Health. 2022 Jan 13;9:789026. doi: 10.3389/fpubh.2021.789026 (PMC8792840; doi:10.3389/fpubh.2021.789026)
Supplement: Supplementary file 2 [file Table_1.docx]

| **Table S1.** Characteristics of patients in external validation cohort | |
| --- | --- |
| **Variable** | **All cohort (n=431)** |
|  | **N (%)** |
| Age (years) |  |
| 65-74 | 280 (65.0%) |
| >74 | 151 (35.0%) |
| Race |  |
| White | 300 (69.6%) |
| Black | 37 (8.6%) |
| Other^a^ | 94 (21.8%) |
| Grade |  |
| I/II | 353 (81.9%) |
| III/IV | 78 (18.1%) |
| T stage |  |
| T1 | 272 (63.1%) |
| T2 | 159 (36.9%) |
| Surgery |  |
| No | 201 (46.6%) |
| Local destruction | 64 (14.8%) |
| Partial resection^c^ | 145 (33.6%) |
| Liver transplantation | 21 (4.9%) |
| Radiotherapy |  |
| No | 381 (88.4%) |
| Yes | 50 (11.6%) |
| Chemotherapy |  |
| No/Unknown | 314 (72.9%) |
| Yes | 117 (27.1%) |
| ^a^Other includes Asian/Pacific Islander, American Indian/Alaskan Native | |
| ^b^Other includes single, unmarried, separated, divorced, widowed and domestic partner | |
| ^c^Partial resection includes wedge resection, segmental resection, lobectomy and extended lobectomy | |
