# Supplementary material for: A Web-Based Prediction Model for Cancer-Specific Survival of Elderly Patients With Early Hepatocellular Carcinoma: A Study Based on SEER Database
Source: Front Public Health. 2022 Jan 13;9:789026. doi: 10.3389/fpubh.2021.789026 (PMC8792840; doi:10.3389/fpubh.2021.789026)
Supplement: Supplementary file 3 [file Table_2.docx]

| **Table S2.** Results of proportional hazard assumption | |
| --- | --- |
| Variable | P |
| Age | 0.99859 |
| Race | 0.98122 |
| Sex | 0.68458 |
| Grade | 0.79070 |
| T stage | 0.45566 |
| Surgery | 0.34215 |
| Radiotherapy | 0.81364 |
| Chemotherapy | 0.17442 |
| Tumor size | 0.29427 |
| Marital status | 0.29484 |
| GLOBAL | 0.481 |
